# Depressive Symptoms during Pregnancy and the Postpartum Period: A Tertiary Hospital Experience

**DOI:** 10.3390/medicina60081288

**Published:** 2024-08-09

**Authors:** Danilo Mladenovic, Sanja Kostic, Katarina Ivanovic, Ivana Jovanovic, Milos Petronijevic, Milica Petronijevic, Svetlana Vrzic Petronijevic

**Affiliations:** 1 Faculty of Medicine, University of Belgrade, 11000 Beograd, Serbia; danilomladenovic16@gmail.com (D.M.); ordinacija.petronijevic@gmail.com (M.P.);; 2 Clinic for Gynecology and Obstetrics, University Clinical Center of Serbia, 11000 Belgrade, Serbia; cara.kostic@gmail.com (S.K.); ikatarina.1996@gmail.com (K.I.); ivana.jovanovic92@yahoo.com (I.J.)

**Keywords:** depression, pregnancy, postpartum, prenatal risk factors

## Abstract

*Background and Objectives*: The prevalence of depressive symptoms during pregnancy is about 20%, and 10–15% in the postpartum period. Suicide is a worrying cause of death among women in these periods. Although ICD-10 lacks specific definitions for perinatal depression (it is planned in ICD-11), the DSM-5 defines it. Various etiological factors and treatment options are being investigated. This study aimed to examine potential etiological factors in order to contribute to potential preventive and therapeutic approaches. *Material and Methods:* A prospective study at the Clinic for Gynecology and Obstetrics, University Clinical Center of Serbia, from October 2023 to January 2024 was conducted. Two hundred and five healthy women were surveyed before giving birth (37+ gestational weeks) and 2 weeks and 2 months after childbirth. The following factors were examined: sociodemographic, psychological, and obstetric (using a specially designed questionnaire); relationship quality (DAS-32); and depression, anxiety, and stress symptoms (EPDS; DASS-21). *Results*: Depression frequency was 26.3% before childbirth, 20% in the second week, and 21.9% in the second month after delivery. DASS-21 test results showed a statistically significant correlation before delivery and two weeks postpartum (*p* = 0.02). Factors that are significantly associated with the presence of depressive symptoms include the following: before childbirth—miscarriages (*p* < 0.01); in the second week after childbirth—personal experiences of a difficult birth (*p* < 0.01), cesarean delivery instead of planned vaginal delivery (*p* = 0.03), and application of epidural anesthesia (*p* = 0.04); and in the second month after childbirth—satisfaction with financial status (*p* = 0.035). Relationship quality is significantly correlated with DASS-21 test results before childbirth, in the second week, and in the second month after childbirth (*p* < 0.01), and it is significantly different in women with and without depressive symptoms (before childbirth, in the second week, and in the second month after childbirth, *p* < 0.01). *Conclusions*: There are risk factors that can be addressed preventively and therapeutically during pregnancy and in labor. This could be achieved through psychotherapy, partner support, and appropriate management of labor.

## 1. Introduction

The prevalence of depressive symptoms during pregnancy is about 20%, and 10–15% in the postpartum period [1,2,3,4,5,6,7]. Suicide is a significant cause of death in women after childbirth [2,8]. In the current International Classification of Diseases and Related Health Problems, 10th Revision (ICD-10), there is no specific definition for depression during pregnancy or postpartum depression. These disorders are classified as a depressive episode if the symptoms align with the general description of a depressive episode, or as a recurrent depressive disorder if there is a history of previous episodes. Postpartum depression symptoms that do not align with the description of a depressive episode are classified as “Mental and behavioral disorders associated with the puerperium” [9]. There are proposals to introduce a category for postpartum depression in the forthcoming ICD-11 [6]. Conversely, the Diagnostic and Statistical Manual of Mental Disorders, Fifth Edition (DSM-5), provides a definition for postpartum depression, requiring the presence of five or more symptoms for diagnosis: negative affect, diminished interest or pleasure in activities, significant weight change (more than 5% in one month), insomnia, psychomotor agitation or retardation, fatigue or loss of energy, feelings of worthlessness or excessive or inappropriate guilt, diminished ability to concentrate, or recurrent thoughts of death or suicidal ideation. DSM-5 criteria specify that symptoms must appear during pregnancy or within the first four weeks postpartum [10]. Various etiological factors are being investigated for the prevention and treatment of depression during pregnancy and postpartum depression [11,12]. These factors can be categorized into the following groups: sociodemographic, psychological, genetic, neuroendocrine, neurotransmitter imbalance, neuroinflammatory, and neuroanatomical [12]. The treatment of these disorders is highly complex due to the need to consider both the physiological and psychological aspects of the mother–child relationship. Additionally, neuroendocrine changes in pregnant women can impact the pharmacodynamics of different therapeutics [13]. Potential treatment options encompass pharmacotherapy, psychotherapy, neuromodulation, hormonal therapy, and some experimental methods [14]. 

This study aimed to examine the association between sociodemographic, psychological, obstetric, and marital factors and depressive symptoms during pregnancy (at 37+ gestational weeks) and two weeks and two months after childbirth, in order to contribute to potential preventive and therapeutic approaches.

## 2. Material and Methods

A prospective study at the Clinic for Gynecology and Obstetrics, University Clinical Center of Serbia in Belgrade, from October 2023 to January 2024, was conducted, involving 205 healthy pregnant women. Excluding criteria in the research were previous psychiatric treatment or usage of psychiatric drugs. Potential factors that could independently or collectively increase vulnerability to developing depression during pregnancy and the postpartum period were examined. These factors were categorized into four groups: sociodemographic, obstetric, psychological, and marital factors.

Sociodemographic factors were collected using a specially designed questionnaire and included data on age, marital status, place of residence, education level, financial status, social support, and whether the pregnancy was desired.

Psychological factors were also assessed with a customized questionnaire. These factors included satisfaction with financial status, perception of the newborn’s future, perceived difficulty of the pregnancy, and perceived difficulty of childbirth.

Obstetric factors were evaluated using a tailored questionnaire for this study, covering the following factors: number of previous deliveries, previous miscarriages and abortions, pregnancy risk (on a scale of 1 to 5), sex of the newborn, if there was pathology of the newborn, multiple pregnancies, planned mode of delivery (cesarean section or vaginal delivery), actual mode of delivery (cesarean section or vaginal delivery), birthweight, head circumference at birth, and method of conception. 

Marital factors were assessed using the Dyadic Adjustment Scale (DAS-32), which comprises 32 items with a score range of 0 to 151 and can be used for both partners. This scale measures marital satisfaction, cohesion, and expression of affection. It has been standardized on the Serbian population with high reliability [15,16,17].

To assess depression and anxiety, the Edinburgh Postnatal Depression Scale (EPDS) and the Depression, Anxiety, and Stress Scale-21 Items (DASS-21) were used. The EPDS, validated for the Serbian population [1,18,19], consists of 10 items that assess the presence of depressive symptoms in postpartum women. The DASS-21 is a 21-item scale measuring depression, anxiety, and stress, representing a shortened version of the original 42-item scale, and is suitable for respondents with lower patience levels. It has demonstrated better psychometric properties and has been validated and translated into Serbian [20]. 

Women were surveyed three times: first at 37+ weeks of gestation, second within the first 2 weeks postpartum, and third at 2 months postpartum. Since both DSM-5 and ICD-10 diagnostic systems require the presence of symptoms for at least 2 weeks to make a diagnosis of a depressive episode, the period of 2 weeks after delivery was chosen to differentiate between “baby blues” effect and postpartum depression [21]. Periods of 37+ weeks of pregnancy and 2 months after delivery were used in accordance with previous studies that used similar time periods, both in Serbian and other populations, in order to obtain information on depressive symptoms during pregnancy and in the postpartum period [1,3,4]. 

Statistical analysis was performed using the EZR: R Commander software (2.7-x). The following statistical tools were employed: Spearman’s rank correlation test, paired *t*-test, two-sample *t*-test, Chi-square test, Fisher’s exact test, Wilcoxon’s signed rank test, and McNemar’s test. The level of significance was set at 0.05.

The study was approved by the Ethics Committee of the University Clinical Center of Serbia (no. 1177/15) on 28 December 2022. All participants provided signed informed consent after being given detailed information about the goals and methods of the research. 

## 3. Results

The average age of participants is 31 years, ranging from 20 to 41 years. The majority of them are married (99%) and live in the city (79.6%). Almost half of them have a higher education level, i.e., a bachelor’s degree (43.3%). More than half of them are primiparous (57.1%). Vaginal delivery was the dominant mode of delivery (74.2%). Data on participant age, marital status, education level, place of residence, parity, and delivery type are presented in Table 1.

The prevalence of depressive symptoms among women was assessed based on EPDS test results with scores of 10 or higher. During the third trimester of pregnancy, the prevalence was 26.3% (54 out of 205 surveyed women). Two weeks postpartum, this percentage decreased to 20% (27 out of 135 who completed the second survey), and it remained at 21.9% in the second month postpartum (21 out of 96 who completed the third survey). There was no statistically significant difference in the prevalence of depressive symptoms before delivery and two weeks postpartum (*p* = 0.37), nor between two weeks postpartum and two months postpartum (*p* = 0.85).

**Figure 1:**
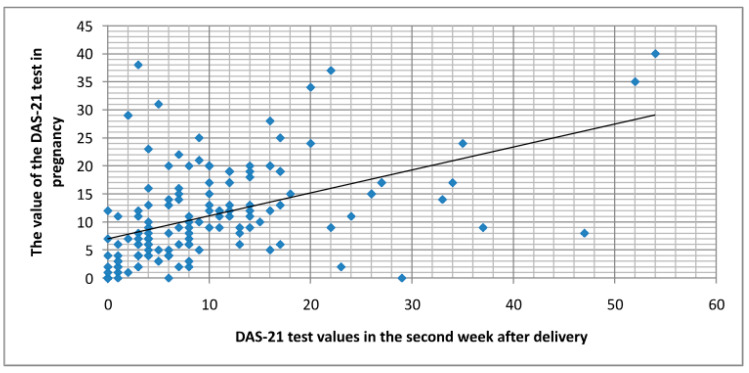
Correlation between DASS-21 test scores before and two weeks after childbirth.

However, the DASS-21 test results, which more comprehensively describe symptoms of depression, stress, and anxiety, showed a statistically significant difference between pre-delivery and two weeks postpartum (*p* = 0.02) (Figure 1), but not between two weeks and two months postpartum (*p* = 0.23).

The presence of depressive symptoms in the first two weeks postpartum showed a statistically significant association with pre-delivery depressive symptoms (*p* < 0.01). Similarly, the presence of depressive symptoms in the second month postpartum was significantly associated with depressive symptoms in the first two weeks postpartum (*p* < 0.01). The correlation between DASS-21 test results in the last trimester and two weeks postpartum was positive (*p* < 0.01), as was the correlation between two weeks postpartum and two months postpartum (*p* < 0.01), as well as the correlation between pre-delivery and two months postpartum (*p* < 0.01).

None of the examined sociodemographic factors (age, place of residence, education level, social support, and whether the pregnancy was desired) showed a statistically significant association with the occurrence of depressive symptoms.

Perception of the difficulty of childbirth emerged as a significant psychological factor associated with depressive symptoms two weeks postpartum (*p* < 0.01) (Table 2). 

Satisfaction with financial status was another significant psychological factor, where women satisfied with their financial status exhibited the lowest incidence of depressive symptoms in the second month postpartum (Table 3). Other examined psychological factors did not show a statistically significant association.

A significant obstetric factor was the history of spontaneous miscarriages (*p* < 0.01) (Table 4). Additionally, women who had planned for a vaginal delivery but underwent a cesarean section showed a statistically significant increase in postpartum depressive symptoms (*p* = 0.03) compared to those who delivered as planned (vaginally or via planned cesarean section) (Table 5). Moreover, the use of epidural anesthesia during childbirth was associated with a lower incidence of depressive symptoms postpartum (Table 5). Other factors such as the baby’s gender, birthweight, pregnancy complications, congenital malformations, and delivery complications did not show a significant association with depressive symptoms.

**Figure 2:**
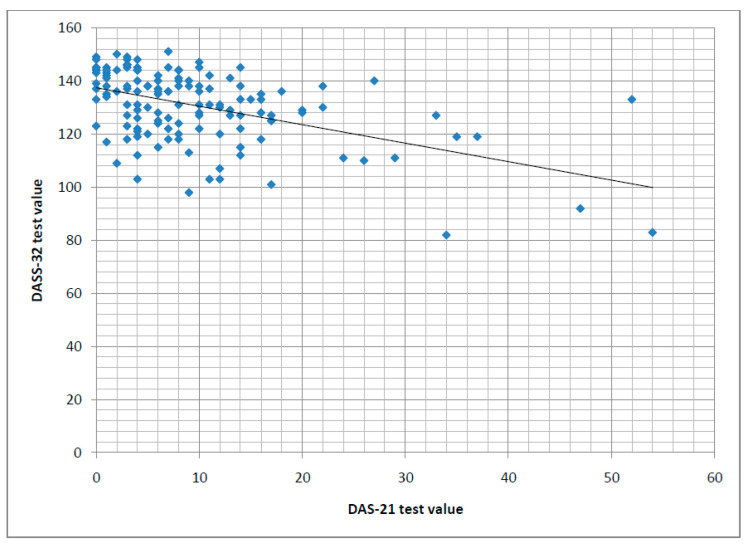
Correlation between values of the DASS-21 test and the DAS-32 test.

The results of the DAS-32 test, which evaluates partner relationships, showed a significant negative correlation with DASS-21 test results, indicating that better partner relationships were associated with lower levels of depression, anxiety, and stress before delivery (*p* < 0.01), two weeks postpartum (*p* < 0.01), and two months postpartum (*p* < 0.01) (Figure 2). Women with depressive symptoms measured by the EPDS test had significantly lower DAS-32 scores compared to those without depressive symptoms, both pre-delivery (*p* < 0.01) and postpartum at two weeks (*p* < 0.01) and two months (*p* < 0.01).

## 4. Discussion

The present study aimed to identify sociodemographic, psychological, obstetric, and marital factors, which could contribute to developing depressive symptoms during pregnancy and in the postnatal period. It has been shown that the frequency of depressive symptoms, measured by the EPDS test, decreased in the postpartum period, although not statistically significantly. However, there was a statistically significant difference in DASS-21 scores before and after childbirth. Dmitrovic et al. reported that 21% of pregnant women in Serbia exhibited depressive symptoms in the third trimester, whereas the prevalence six weeks after childbirth was 11% [1]. This is lower than our findings of 26.3% during pregnancy, 20% in the second week postpartum, and 21.9% in the second month postpartum. Globally, Li et al. reported a postpartum depression prevalence of 17.3% at two months postpartum [3], which is less than our finding. Poorandokht et al. reported a higher prevalence of 38.8% between two weeks and six months postpartum [4], while Shi et al. reported 14.6% at six weeks postpartum [6]. 

Prenatal depressive symptoms were identified as statistically significant factors for postpartum depressive symptoms, corroborating previous research [1]. Therefore, prenatal and postpartum depressive symptoms cannot be viewed independently. Additionally, a history of depressive episodes throughout life is identified as a risk factor [2,4,6,7].

Satisfaction with financial status is a psychological factor significantly associated with postpartum depressive symptoms in previous research both in Serbia [1] and other populations [5,22], as confirmed in this study. Some studies measured financial status itself; however, our study included personal perception of financial status. Personal experience of difficulty during childbirth is also a significant factor associated with postpartum depressive symptoms, which is a factor not previously studied in Serbia [1].

Obstetric factors that should be highlighted include prior miscarriages and changes from planned vaginal delivery to cesarean section. Previous research has already noted the negative impact of depression during pregnancy on pregnancy progression, fetal development, and future neonatal development [23,24,25]. Poorandokht et al. reported an association between depressive symptoms, congenital anomalies, and increased hospitalization rates in newborns [4]. Our study found a significant correlation between prior miscarriages and depressive symptoms during pregnancy. It raises the question of whether women with prior miscarriages also exhibited depressive, anxiety, or stress symptoms that could have led to the miscarriage. This warrants further investigation. Additionally, the significant association between depressive symptoms in the early postpartum weeks and unplanned cesarean sections indicates an interrelation between depressive symptoms and delivery method. Zareba et al. also noted the correlation between depressive symptoms and complications during childbirth, such as blood loss over 1000 mL and prolonged second and third stages of labor [7]. Previous studies have shown different results regarding the impact of epidural anesthesia. Edipoglu et al. found no significant association in the prevalence of depressive symptoms between groups who received and did not receive epidural anesthesia during childbirth [26], whereas Tan et al. found this association significant, which is consistent with our findings [27]. Further research is needed to draw more definitive conclusions.

Partner relationships, measured by the DAS-32 test, showed a significant correlation with DASS-21 test results, which measure levels of depressive symptoms, anxiety, and stress. As DAS-32 scores, indicating better partner relationships, increase, DASS-21 scores decrease, indicating lower levels of depressive symptoms, anxiety, and stress. This highlights the critical influence of partner relationships on the development of perinatal depressive symptoms. Earlier research showed a connection between partner support levels and the development of depressive symptoms [3]. 

The findings from the present study indicate that certain factors, such as poor partner relationships, low socioeconomic status, negative psychological perception of pregnancy and childbirth, cesarean section, and previous spontaneous miscarriages, are associated with a higher prevalence of depressive symptoms before and after childbirth. However, study limitations primarily pertain to the compliance of respondents participating in the study. Initially, 205 women participated in the prepartum survey. This number decreased to 135 women who agreed to participate in the second survey (two weeks postpartum), indicating a 35.15% dropout rate. The third survey (two months postpartum) was completed by 97 women, which is 28.15% less than the second survey and 52.68% less than the prepartum survey. Nevertheless, since the study was conducted in a tertiary institution, the presented results are a realistic representation of perinatal depression in the Serbian population. 

## 5. Conclusions

Depressive symptoms during pregnancy and the postpartum period represent a significant socio-medical issue. It is important to bear in mind that the pregnancy and postpartum periods cannot be taken into consideration independently. 

Women exhibiting factors that are statistically significantly associated with depressive symptoms during pregnancy and the postpartum period, as identified in this study, should receive psychological and psychiatric support to mitigate the negative impact of these symptoms on both mother and child. Partner support and the appropriate management of labor could be an important modality for reducing depressive symptoms, stress, and anxiety, in both pregnant and postpartum women. The appropriate time for intervention is certainly during pregnancy, given the connection between depressive symptoms before and after childbirth. Couples therapy can be implemented according to the DAS-32 results with a focus on the relationship segment (satisfaction, cohesion, and expression of affection) that is causing a lower score on this scale. If the expression of affection part of the scale is lower, there are emotion-focused therapies that can improve this segment of the relationship, thus lowering the chance of depressive symptom appearance during pregnancy and the postpartum period. Also, various types of cognitive therapy can be used during pregnancy for managing labor, focusing on anxiety and depressive thoughts with the purpose of restructuring them and disrupting destructive thought patterns. Further research in clinical practice is needed to evaluate the effectiveness of these therapeutic modalities.

## Figures and Tables

**Table 1:** Demographic and obstetric factors of the participants.

Age (years)					
Mean	Min		Max		Median
31	20		41		31
Marital status					
Married			Single		
99%			1%		
Education level					
Primary school			1%		
High school			36.9%		
Bachelor’s degree			43.3%		
Master			15.3%		
PhD			3.4%		
Place of residence					
City		Suburb		Village	
79.6%		12.9%		7.5%	
Parity					
Primiparous			Multiparous		
57.1%			42.9%		
Delivery type					
Vaginal			Cesarean section		
74.2%			25.8%		

**Table 2:** The frequency of depressive symptoms in the second week after childbirth depending on the personal perception of childbirth difficulty.

Personal Experience of the Difficulty of Childbirth				
Depression Symptoms	Easy	Medium	Hard	Chi-Square Test Value
yes	7	7	13	*p* < 0.01
no	53	40	15	

**Table 3:** Frequency of depressive symptoms in second month after childbirth depending on satisfaction with financial status.

Satisfaction with Financial Status				
Depression Symptoms	Unsatisfied	Medium Satisfied	Satisfied	Fisher’s Exact Test Value
yes	4	6	10	*p* = 0.035
no	7	12	57	

**Table 4:** Frequency of depressive symptoms during pregnancy (37+ gestational weeks) depending on previous miscarriage.

Previous Miscarriage			
Depression Symptoms	Yes	No	Chi-Square Test Value
yes	26	28	*p* < 0.01
no	32	119	

**Table 5:** The frequency of depressive symptoms in the second week after delivery depending on the use of epidural anesthesia during delivery and the change from a planned vaginal delivery to a cesarean delivery.

Epidural Anesthesia during Delivery			
Depression Symptoms	Yes	No	Chi-Square Test Value
yes	6	18	*p* = 0.04
no	46	48	
Planned Vaginal Delivery was Completed by Cesarean Section			
Depression Symptoms	Yes	No	Chi-Square Test Value
yes	8	19	*p* = 0.03
no	12	96	

## Data Availability

The original contributions presented in the study are included in the article, further inquiries can be directed to the corresponding authors.
